# A transfer learning approach to facilitate ComBat-based harmonization of multicentre radiomic features in new datasets

**DOI:** 10.1371/journal.pone.0253653

**Published:** 2021-07-01

**Authors:** Ronrick Da-ano, François Lucia, Ingrid Masson, Ronan Abgral, Joanne Alfieri, Caroline Rousseau, Augustin Mervoyer, Caroline Reinhold, Olivier Pradier, Ulrike Schick, Dimitris Visvikis, Mathieu Hatt

**Affiliations:** 1 INSERM, UMR 1101, LaTIM, University of Brest, Brest, France; 2 Radiation Oncology Department, University Hospital, Brest, France; 3 Department of Radiation Oncology, Institut de cancérologie de l’Ouest René-Gauducheau, Saint-Herblain, France; 4 Department of Nuclear Medicine, University of Brest, Brest, France; 5 Department of Radiation Oncology, McGill University Health Centre, Montreal, Quebec; 6 Department of Nuclear Medicine, Institut de cancérologie de l’Ouest René-Gauducheau, Saint-Herblain, France; 7 Department of Radiology, McGill University Health Centre, Montreal, Canada; 8 Augmented Intelligence & Precision Health Laboratory of the Research Institute of McGill University Health Centre, Montreal, Canada; Universita degli Studi di Perugia, ITALY

## Abstract

**Purpose:**

To facilitate the demonstration of the prognostic value of radiomics, multicenter radiomics studies are needed. Pooling radiomic features of such data in a statistical analysis is however challenging, as they are sensitive to the variability in scanner models, acquisition protocols and reconstruction settings, which is often unavoidable in a multicentre retrospective analysis. A statistical harmonization strategy called ComBat was utilized in radiomics studies to deal with the “center-effect”. The goal of the present work was to integrate a transfer learning (TL) technique within ComBat—and recently developed alternate versions of ComBat with improved flexibility (M-ComBat) and robustness (B-ComBat)–to allow the use of a previously determined harmonization transform to the radiomic feature values of new patients from an already known center.

**Material and methods:**

The proposed TL approach were incorporated in the four versions of ComBat (standard, B, M, and B-M ComBat). The proposed approach was evaluated using a dataset of 189 locally advanced cervical cancer patients from 3 centers, with magnetic resonance imaging (MRI) and positron emission tomography (PET) images, with the clinical endpoint of predicting local failure. The impact performance of the TL approach was evaluated by comparing the harmonization achieved using only parts of the data to the reference (harmonization achieved using all the available data). It was performed through three different machine learning pipelines.

**Results:**

The proposed TL technique was successful in harmonizing features of new patients from a known center in all versions of ComBat, leading to predictive models reaching similar performance as the ones developed using the features harmonized with all the data available.

**Conclusion:**

The proposed TL approach enables applying a previously determined ComBat transform to new, previously unseen data.

## Introduction

The extraction of quantitative features using high-throughput computing from medical images like magnetic resonance [MR], computed tomography [CT], and positron emission tomography [PET], is known as radiomics [1–4]. It provides a large set of quantitative features to researchers, enabling investigation of potential impact in clinical-decision support systems to improve diagnostic, prognostic, and predictive accuracy [5]. These various radiomics-driven prognostic/predictive studies in various cancer types may prove useful for personalized medicine in oncological applications [6].

The increased interest in radiomics research is in part due to the transparency of radiomics-based models. Thus, many initiatives have recognized the need for greater standardization of radiomics research with the aims of achieving improved reproducibility and translation of radiomics research into clinical practice [7–9]. Despite the significant impact in clinical practice, most radiomics studies to date have been single center based and retrospective in nature, and most published models have not been externally validated [10,11]. In the interest of producing convincing results with respect to the potential clinical value of radiomics as a prognostic tool, it is vital to consider large patient cohorts that can often only be available through multicenter recruitment [12–15]. One of the most important advantages of multicenter studies is the higher statistical relevance and potential generalizability of the developed models when applied to external, previously unseen cohorts. Besides the legal, ethical, administrative and technical hurdles of collecting data from several centers, one of the most challenging aspects is the fact that medical images have different characteristics when acquired on different scanner models from various manufacturers, using different acquisition protocols and reconstruction settings, which is currently unavoidable in the current clinical practice. Radiomic features have been shown to exhibit sensitivity to such heterogeneity, which consequently hinders pooling data to perform statistical analysis and/or machine learning (ML) in order to build robust models [16–21]. We recently reviewed and discussed the existing methods to perform data integration either by harmonizing images before feature extraction, or directly in the already extracted radiomics features by statistically estimating and reducing the unwanted variation associated with center effects [20,22]. In the present work, we place ourselves in the context of features harmonization (i.e., the original images are not specifically pre-processed for harmonization).

In this context, various methods have been considered [20,22]. We have recently expanded the ComBat method to improve its flexibility and robustness [23]. One remaining limitation of ComBat lies in its ability to harmonize previously unseen data (either new patients from one of the centers included in the initial harmonization process, or a new cohort from an entirely new and unseen center) [20]. In this case, the new data has to be labeled and added to the previously considered datasets, and the entire new datasets has to be re-harmonized again, which is cumbersome and seriously hinders the future external validation of the models that have been developed on harmonized features, especially if original features are not available anymore.

In this work, our objective was to develop and evaluate a transfer learning (TL) technique implemented within ComBat (and B(M)-ComBat versions as well) that could allow applying the previously learned harmonization transform to the radiomic features values of new patients from a known center.

## Material and methods

### ComBat approach description

The ComBat strategy was initially drafted for genomics [24], where the so-called “batch effect” is the source of variations in measurements caused by handling of samples by different laboratories, tools and technicians. This “batch effect” is theoretically similar to variations induced in radiomic features by the scanner model, the acquisition protocol and/or the reconstruction settings, sometimes called “center effect”.

ComBat is primarily based on an empirical Bayes framework to eliminates batch effects. It has shown robustness with small sample sizes, down to 5 samples per batch [22,25,26], and continues to be a widely used approach [20,27–29]. ComBat was seen as being “*best* ready *to lessen and remove batch effects while expanding precision and accuracy*” when compared to five other popular batch effect removal methods [20,25]. Within the context of radiomic features harmonization, ComBat works with the following steps:

### Step 1: Standardize the data

The magnitude of radiomic features could differ across center due variability in scanner models, acquisition protocols and reconstruction settings. If not accounted for, these will create bias in the Empirical Bayes (EB) estimates of the prior distribution of center effect and reduce the amount of systematic center information that can be borrowed across features [24]. To avoid this phenomenon, we first standardize the data features-wise so that radiomic features have similar overall mean and variance. Ordinary least-squares is used to calculate features-wise mean and standard deviation estimates, ${\hat{\alpha }}_{g}$ and ${\hat{\sigma }}_{g}$, across feature *g*, sample *j*, and center *i*. The standardized set of features *Z*_*ijg*_ from the original set of features *Y*_*ijg*_ is given by

$$Z_{ijg}=\frac{Y_{ijg}-{\hat{\alpha }}_{ig}-X{\hat{\beta }}_{g}}{{\hat{\sigma }}_{ig}}$$ (1)

where ${X\hat{\beta }}_{g}$ represents potential non-center related covariates and coefficients in the model.

### Step 2: EB center effect parameter estimates using parametric empirical priors

The standardized data is assumed to be normally distributed $Z_{ijg}\sim N(\gamma_{ig},\delta_{ig}^{2})$.

Additionally, we assume the parametric forms for prior distributions on the center effect parameters to be

$$\gamma_{ig}\sim N(Y_{i},\tau_{i}^{2})\;\text{and}\;\delta_{ig}^{2}\sim Inverse\;Gamma\;(\lambda_{i},\theta_{i})$$ (2)

The hyperparameters *γ*_*i*_, $\tau_{i}^{2}$, *λ*_*i*_, *θ*_*i*_ are estimated empirically from standardized data using the method of moments. These prior distributions (Normal, Inverse Gamma) were selected due to the conjugacy with the Normal assumption for the standardized data [24]. Based on the distributional assumptions above, the EB estimates for center effect parameters, *γ*_*ijg*_ and $\delta_{ig}^{2}$ are given (respectively) by the conditional posterior means

$$\gamma_{ig}^{*}=\frac{n_{i}{\bar{\tau }}_{i}^{2}{\hat{\gamma }}_{ig}+\delta_{ig}^{2*}{\bar{\gamma }}_{i}}{n_{i}{\bar{\tau }}_{i}^{2}+\delta_{ig}^{2*}}\;\text{and}\;\delta_{ig}^{2*}=\frac{{\bar{\theta }}_{i}+\frac{1}{2}\sum_{j}{\left(Z_{ijg}-\gamma_{ig}^{*}\right)}^{2}}{\frac{n_{j}}{2}+{\bar{\lambda }}_{i}-1}$$ (3)

Detailed derivations for these estimates *γ*_*ig*_ and $\delta_{ig}^{2}$ are given in the supplemental materials available at *Biostatistics* online.

### Step 3: Adjust the data for center effects

After calculating the adjusted center effect estimators, $\gamma_{ig}^{*}$ and $\delta_{ig}^{2*}$, we now adjust the data. The EB center-adjusted data $y_{ig}^{*}$ is given by

$$Y_{ijg}^{*}=\frac{{\hat{\sigma }}_{g}}{{\hat{\delta }}_{ig}^{*}}(Z_{ijg}-{\hat{\gamma }}_{ig}^{*})+{\hat{\alpha }}_{g}+X{\hat{\beta }}_{g}$$ (4)

Although ComBat is an effective method, one of its limitations is that it centers the data to the overall, grand mean of all samples, resulting in an adjusted data matrix that is shifted to an arbitrary location that no longer coincides with the location of any of the original centers. This can result in harmonized features losing their original physical meaning, including the generation of impossible values, *e*.*g*., negative volumes or SUV.

Recently, we showed the interest of two modifications [20,30]: on the one hand, a first modification, called M-ComBat, allows centering the data to the location and scale of a pre-determined “reference” batch, which, in the case of radiomics, prevents losing the physical meaning of some features (e.g., SUV or volume) and can provide the ability to select as a reference a dataset for which confidence in data curation is higher [30]. Bootstrapped ComBat (B-ComBat) on the other hand, improves the predictive ability of the developed models and their robustness through the addition of a bootstrap step [20]. These improvements however did not address the limitations of ComBat regarding the application of models based on harmonized features on new patients.

#### M-ComBat

M-ComBat shifts samples to the mean and variance of the chosen reference batch, instead of the grand mean and pooled variance [20,30]. This is accomplished by changing the standardizing mean and variance of the estimates, ${\hat{\alpha }}_{g}$ and ${\hat{\sigma }}_{g}$, to center-wise estimates, ${\hat{\alpha }}_{ig}$ and ${\hat{\sigma }}_{ig}$. Moreover, the mean and variance estimates utilized in the final center-effect adjusted data are calculated using the feature-wise mean and variance estimates of the reference batch, *i* = *r*.

The M-ComBat adjusted data are then given by

$$Y^{*}{}_{ijg}=\frac{{\hat{\sigma }}_{i=r,g}}{{\hat{\delta }}^{*}{}_{ig}}(Z_{ijg}-{\hat{\gamma }}^{*}{}_{ig})+{\hat{\alpha }}_{i=r,g}+X{\hat{\beta }}_{g}$$ (5)

#### Bootstrapped ComBat: B-ComBat and BM-ComBat

Our last study [50] showed the interest of a hybrid technique performing parametric bootstrap in the initial estimates obtained in ComBat (or M-ComBat), then use a Monte Carlo method to obtain the final estimates. Hence, the final B-ComBat and BM-ComBat bootstrapped adjusted data are given respectively by:

$$Y_{ijg}^{B–ComBat}=\frac{y_{ijg}-{\hat{\alpha }}_{gk}-X_{ij}{\hat{\beta }}_{gk}-\gamma_{igk}^{*}}{\delta_{igk}^{*}}+{\hat{\alpha }}_{gk}+X_{ij}{\hat{\beta }}_{gk}$$ (6)

$$Y_{ijg}^{BM–ComBat}=\frac{y_{ijg}-{\hat{\alpha }}_{i=(r,g)k}-X_{ij}{\hat{\beta }}_{gk}-\gamma_{igk}^{*}}{\delta_{igk}^{*}}+{\hat{\alpha }}_{i=(r,g)k}+X_{ij}{\hat{\beta }}_{gk}$$ (7)

#### Proposed method: A transfer learning (TL) approach

We first determine the hyper parameters, *i*.*e*., the conditional posterior center effect estimators ($\gamma_{ig}^{*}$ *and $\delta_{ig}^{2*}$*) in the initial available dataset (“learning” part). Then, these learned hyper parameters are used in the harmonization process of the new, previously unseen dataset (“transfer” part).

The proposed method follows these steps:

- *Save the conditional posterior center effect estimators ($\gamma_{ig}^{*}$ and $\delta_{ig}^{2*}$) obtained in* ***Step 2*** *during the initial data harmonization using ComBat*.
- *Perform the* ***Step 1*** *using the new unseen data*.
- *After obtaining the results of* ***(i)*** *and* ***(ii)***, *perform* ***Step 3*** to adjust the new unseen data. The new EB center-adjusted data $y_{ijg}^{TL}$ is given by

$$Y_{ijg}^{TL}=\frac{{\hat{\sigma }}_{g}}{{\hat{\delta }}_{ig}^{*}}(Z_{ijg}-{\hat{\gamma }}_{ig}^{*})+{\hat{\alpha }}_{g}+X{\hat{\beta }}_{g}$$ (8)

### Data: Patient cohorts, imaging and clinical endpoints

In this study, we relied on a dataset of 189 patients with histologically proven locally advanced cervical cancer (LACC) retrospectively collected from three clinical centers (Brest, n = 117 and Nantes, n = 44, in France, and Montreal, n = 28, in Canada). Patients were treated with definitive curative chemoradiotherapy followed by brachytherapy from August 2010 to July 2017 (to ensure a minimum follow-up of 1 year) (see S1 Table). The radiomics analysis was applied to the available pre-treatment images: T2-weighted MRI (T2) and apparent diffusion coefficients (ADC) maps from diffusion-weighted MRI, post-injection gadolinium contrast-enhanced MRI (GADO), and Fluorodeoxyglucose (FDG)-PET images (see S2 Table). Importantly, the PET/CT settings (scanner model, reconstruction algorithms and parameters) were the same within Brest and Nantes, but not within Montreal, where 2 different scanners were used for 5 and 23 patients respectively (see S1 Table). Compared to our previous work [20] in which 50 patients from Nantes were included, 6 were removed for the present analysis because their PET images had different characteristics. The available clinical variables included age (gender is female for all patients), histopathological type, grade, lymphovascular invasion, HPV status, T-stage, N-stage and FIGO (International Federation of Gynaecology and Obstetrics). To provide a rationale to adapt treatment (*e*.*g*., avoid systemic treatment for patients with low risk of recurrence), prediction of local failure (LF) was chosen as the endpoint [20,31].

All procedures performed in studies involving human participants were in accordance with the ethical standards of the institutional and/or national research committee and with the 1964 Helsinki declaration and its later amendments or comparable ethical standards. The retrospective collection of images and clinical data from the three centers was approved by the following ethics committees: "The Institutional Review Board of Brest University Hospital" for data collected in Brest and Nantes, and « The McGill University Health Center Research Ethics Board" for these collected in McGill. All patients provided permission for the use of their clinical data for scientific purposes and informed consent for the anonymous publication of data via a non-opposition form. Data were anonymized before it was accessed for the present analysis.

### Overview of the framework of radiomics analysis

As in our previous work [20], we worked with radiomic features extracted from the images and the available clinical factors collected during the study by Lucia, et al [15]. These are made available for reproducibility. As a reminder, we summarize the process followed by Lucia, et al in the following. More details can be found in [15]. One single expert radiation oncologists (F. Lucia) semi-automatically delineated tumour volume-of-interests (VOIs) independently in the PET and MRI images. The Fuzzy locally adaptive Bayesian (FLAB) algorithm [32] implemented in a home-made software (MIRAS software v1.0.2.0, LaTIM, INSERM, Brest) was used in PET images while the 3D Slicer^™^ software [33] was used in MR images. For each VOI in the PET and three MRI sequences, 79 morphological and intensity-based features, as well as 94 textural features were extracted in 3D using MIRAS software. Features were checked for consistency with the benchmark of the Image Biomarker Standardization Initiative (IBSI) [34,35]. Both “fixed bin number” (FBN, 64 bins) and “fixed bin width” (FBW, width of 0.25, 0.5, 1 and 2 standardized uptake values (SUVs) for PET and width of 10 mm^2^/s for ADC map) grey-level discretization algorithms were used to compute each of the 94 textural features. Texture matrices were built according to the merging procedure (by summation of 13 matrices calculated in toward every direction before texture calculation).

### Experimental analysis

Non-parametric versions of ComBat were utilized in sections A, B, C and D using as harmonization labels either the 3 clinical centers for MRI features and the 4 scanners (1 in Brest, 1 in Nantes and 2 in Montreal) for FDG PET.

The objective of our the experiment is to demonstrate the ability of the TL implementation within ComBat to successfully harmonize features of new patients, previously not included in the initial harmonization.

**Fig 1:**
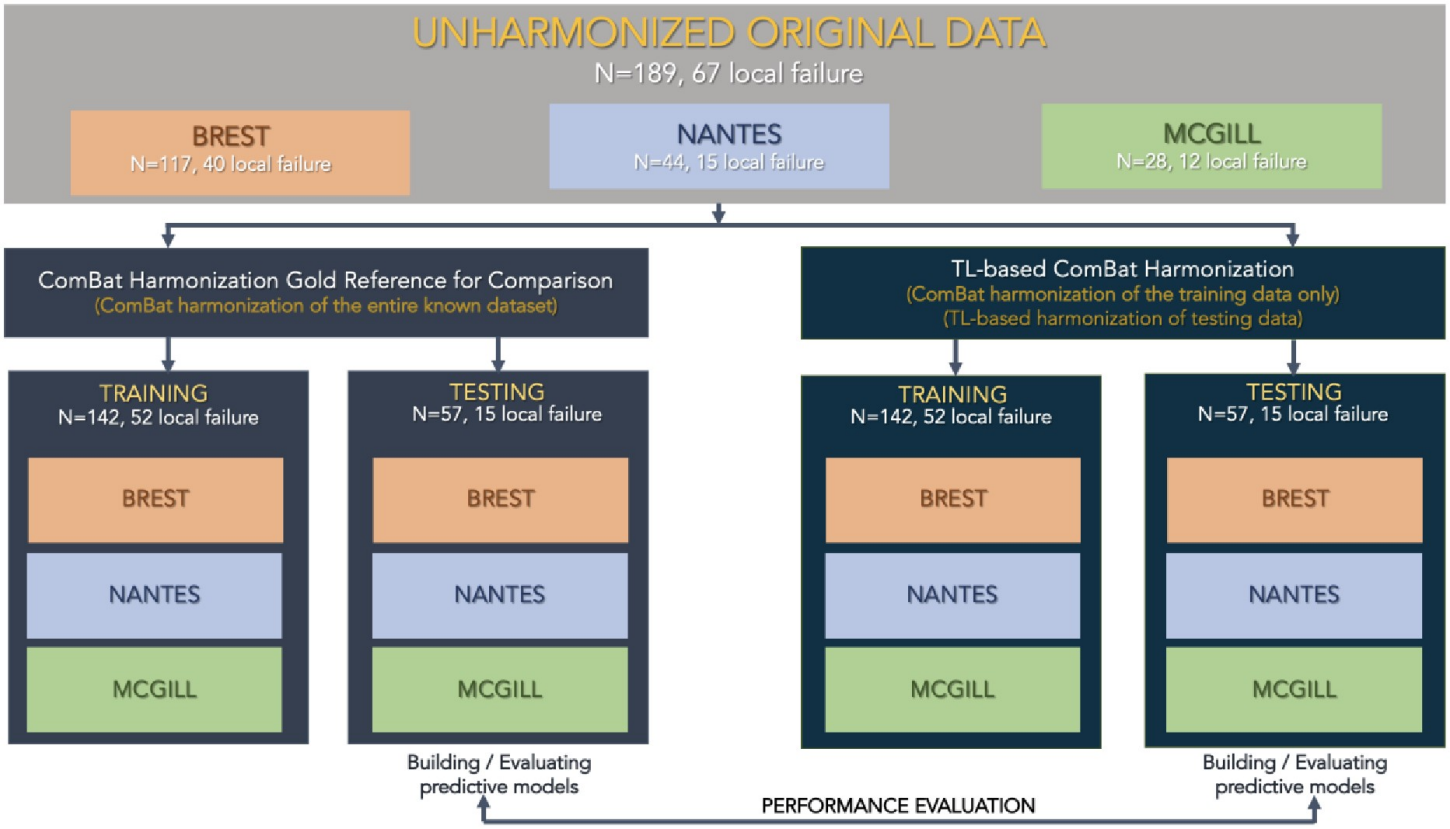
Workflow for the analysis in LACC datasets’ experiments.

Stratified random sampling was used to split the data from the 3 centers into training (n = 142 with 52 LF) and testing (n = 57 with 15 LF) sets. These sets are considered “training” and “testing” for the purpose of building multiparametric models predictive of LF using three different machine learning pipelines (see the next section). Patients from the 3 centers are thus included in both sets. For the purpose of evaluating the TL ComBat harmonization, patients from the testing set are not used in the initial harmonization, and are used to evaluate the performance of the TL harmonization. In order to evaluate the performance of TL ComBat harmonization, all patients from Nantes are set aside in the initial harmonization process and constitute the “new” center to evaluate TL harmonization. The experiment is further illustrated in Fig 1.

In order to further evaluate the impact of harmonization, principal components analysis (PCA) was performed, and the four different versions of ComBat were then compared with ANOVA in terms of their statistical distributions across labels (i.e., 3 clinical centers for MRI features and 4 scanners (1 in Brest, 1 in Nantes and 2 in Montreal) for FDG PET) before and after harmonization with the four ComBat versions. In addition, a 2-sample Kolmogorov-Smirnov test was used to determine if there was a significant difference in the distribution of the features from each device variations both before and after ComBat harmonization.

The Original (i.e., original harmonized data with all the available samples, thus the “gold standard reference”) was compared to the harmonized data by the proposed TL (i.e., harmonized data by the proposed transfer learning) method. Since variances of the results acquired by the two experiments could not be considered equal, we used the Welch-t test [26] to compare whether the differences between the original (i.e., gold standard) and the TL results were statistically different. Tests were run on each combination of data under the null hypotheses “method does not impact ML performances” and “both methods have same performances” and reject the Ho if p < 0.05.

Finally, the performance of the predictive models built relying on features harmonized with the TL approach (through all four ComBat versions) was compared to the performance of the models built using features harmonized using all the available data.

Models predicting endpoints (as a binary task) were built exactly as in our previous work: 3 different ML pipelines were utilized: i) Random Forest (RF) and ii) Support Vector Machine (SVM), both with embedded feature selection, and iii) Multivariate regression (MR) with 10-fold cross-validation after feature selection based on least absolute shrinkage and selection operator (LASSO). All of the harmonized (with the 4 ComBat versions) radiomic features were used as inputs in combination with the available clinical factors (age, gender, histology, stage, etc.). Since there were ~34% of events (LF), we used synthetic minority over-sampling technique (SMOTE) to facilitate training of the models [20,36].

**Fig 2:**
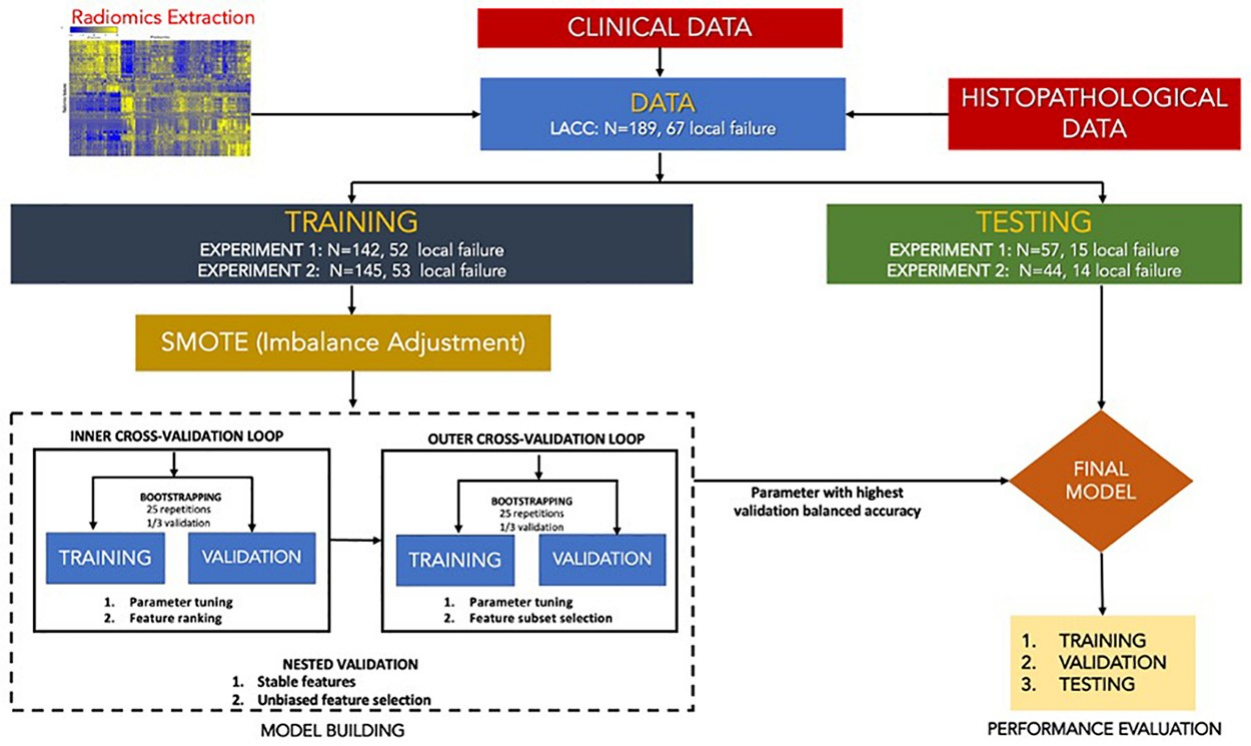
Overall workflow for the analysis in LACC datasets.

For the purpose of the M-ComBat and BM-ComBat, Brest was chosen as the reference to which the two other centers (in the case of MRI) or 3 other scanners (in the case of PET) were harmonized. Fig 2 illustrates the overall workflow.

### Machine learning methodology

#### Imbalance adjustment

To address the imbalance in the dataset, SMOTE algorithm was utilized to facilitate training of the models. SMOTE is a method of over-sampling the minority class in order to provide a balanced number of positive and negative cases to the learning algorithm [20,36]. The difference of SMOTE to any other techniques is that the minority class is over-sampled by creating a synthetic sample rather than over-sampling with replacement [20,36].

#### Multivariate regression with LASSO

Features selected from LASSO was using for training multivariate regression. Here, LASSO was used as both a regularization and variable selection methods for any statistical models [20,37]. LASSO was used to penalize the negative log of the partial likelihood in multivariate cox regression [37]. The algorithm employs a cyclical coordinate descent, which sequentially optimizes the objective function over a parameter with others kept fixed, and cycles repeatedly until convergence [20,37].

#### Random forest

The RF method is designed to make use of an ensemble method consisting of many decision trees [20,38]. The concept behind RF is that each decision tree is formed by choosing the sample from the original dataset with the bootstrap method and selecting the random number of all variables in every decision node. The RF strategy consists of the following steps: i.) n features are randomly selected from a total of m features, ii.) the node d the used to best split point is calculated using the n features, iii.) it checks whether the number of final nodes reaches the target number, and iv.) by repeating step i to iii for n times, a forest is then built [20,38].

#### Support vector machine

SVM is a supervised learning algorithm was incorporated based on a statistical learning theory [39]. It works by aiming to find the hyper-plane, which separates classes from each other, and which is the most distant from both classes. The result is a linearly separable dataset made by using a kernel function [20,39]. Also, a non-linear separation can be made, and the data can be separated in the high dimensions [39] which sometimes resulted to over-fitting in the input space. Overfitting is controlled through the principle of structural risk minimization [20,39].

#### Feature selection methods

The objective of feature selection is to improve the prediction performance of the predictors, understand the underlying process that generated the data and in most of the cases to provide faster and more cost-effective predictors. Given a training data set consisting of N instances, P predictor variables/features X_i_ (i = 1,…, P) and the class Y in {1, 2,…, C}, the objective of feature selection is to select a compact variable/feature subset without loss of predictive information about Y. Feature selection were embedded in both RF and SVM as a part of the model training process and hyper parameters optimization. It is in this manner typically specific to a given learning algorithm, *i*.*e*., the feature subset selection can be considered as a search in the combined space of feature subsets and hypotheses [20,40]. Regarding RF, a single decision partitions the input space into a set of disjoint regions, and assigns a response to each corresponding region [20,40]. In the event of SVM, a similar procedure was thought of in spite of the fact that, rather than the measure of variable importance as in RF, features are ranked based on the best fine cost of the models and are ranked according to the values of leave-one-out error (LOO−*i*), *i*.*e*., the feature *i* with the highest value of LOO−*i* is ranked first [20,41].

#### Final model construction and evaluation

Multiple regression with LASSO, RF and SVM models were fitted independently with the selected optimal features subset and parameters to exploit the feature selection and parameter tuning results.

For classification problem, the discrimination evaluation of the optimal solution during the training can be defined on different performance matrix. Sensitivity, Specificity and accuracy results are provided in the S1–S3 Figs. We have decided to focus on three metrics, area under the ROC curves (AUC), balanced accuracy (BAcc) and Matthew’s correlation coefficient (MCC, worst value = -1; best value = +1) [20,42] to compare the results obtained without and with the different ComBat versions. ROC-AUC measure the optimal learning model underneath the ROC curve which AUC values reflects the overall ranking performance of a classifier based on thresholding settings. BAcc (calculated as the average of sensitivity and specificity) is a more appropriate metric in the presence of data imbalance than the conventional accuracy. Lastly, MCC is a contingency matric method calculating the Pearson product-moment correlation coefficient between the actual and predicted and is a good metric to measure the quality of the binary classification.

## Results

### Initial analysis

The COV measurements (Table 1) show that in the testing sets of the experiment, the TL data exhibit similar variability in all ComBat-harmonized versions (slightly lower/higher in ComBat and B-ComBat, slightly higher/lower in M-ComBat and BM-ComBat) as the one observed in harmonization carried out using all the data.

**Table 1:** COV computed on the Orig and TL data in four ComBat versions.

Data	COV	
	Original	TL
Untransformed	2833	2833
ComBat	1313	1159
B-ComBat	1290	1082
M-ComBat	1204	1309
BM-ComBat	1189	1210

Original = original harmonized data (gold standard reference), TL = harmonized data by the proposed transfer learning method.

According to ANOVA, 97% and 98% (in *Orig* and *TL*, respectively) of untransformed radiomic features were significantly (at p<0.01 level) different between labels. After harmonization, all of the four ComBat versions (in both Orig and TL) completely eliminated significant label related differences across the different cohorts in both datasets, *i*.*e*., none of the radiomic features remained significantly (at p<0.01 level) different between labels.

Table 2 confirms that any ComBat versions and the untransformed data have a significant difference in data distribution. However, for both experiments 1 and 2, the four ComBat versions in the Original and TL, respectively have similar data distributions.

**Table 2:** P-values of Kolmogorov-Smirnov comparing different 4 ComBat versions using Original and TL data.

Kolmogorov-Smirnov				
Untransfomed *vs*. any ComBat	Original *vs*. Transfer Learning			
	ComBat	B-ComBat	M-ComBat	BM-ComBat
<0.0001	0.753	0.921	0.977	0.992

**Fig 3:**
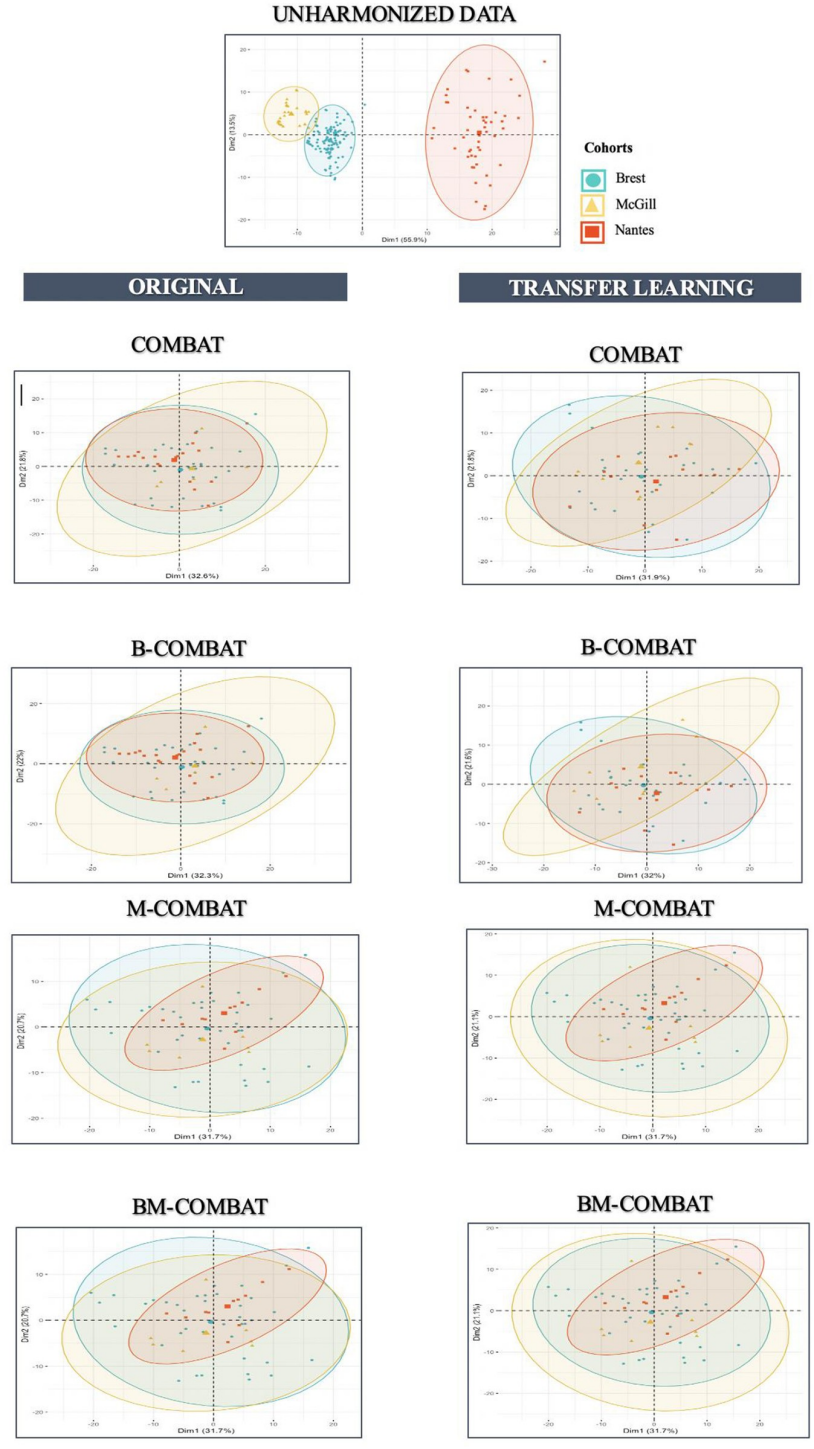
PCA and summary distribution in Experiment 1 in MRI. Scatter plots of top 2 principal components of the radiomic features across the three labels (centers) using data transformed with the 4 versions of ComBat (using R (3.5.1) and R Studio (1.1.456,R Studios Inc., Boston,MA) https://cran.r-project.org/).

**Fig 4:**
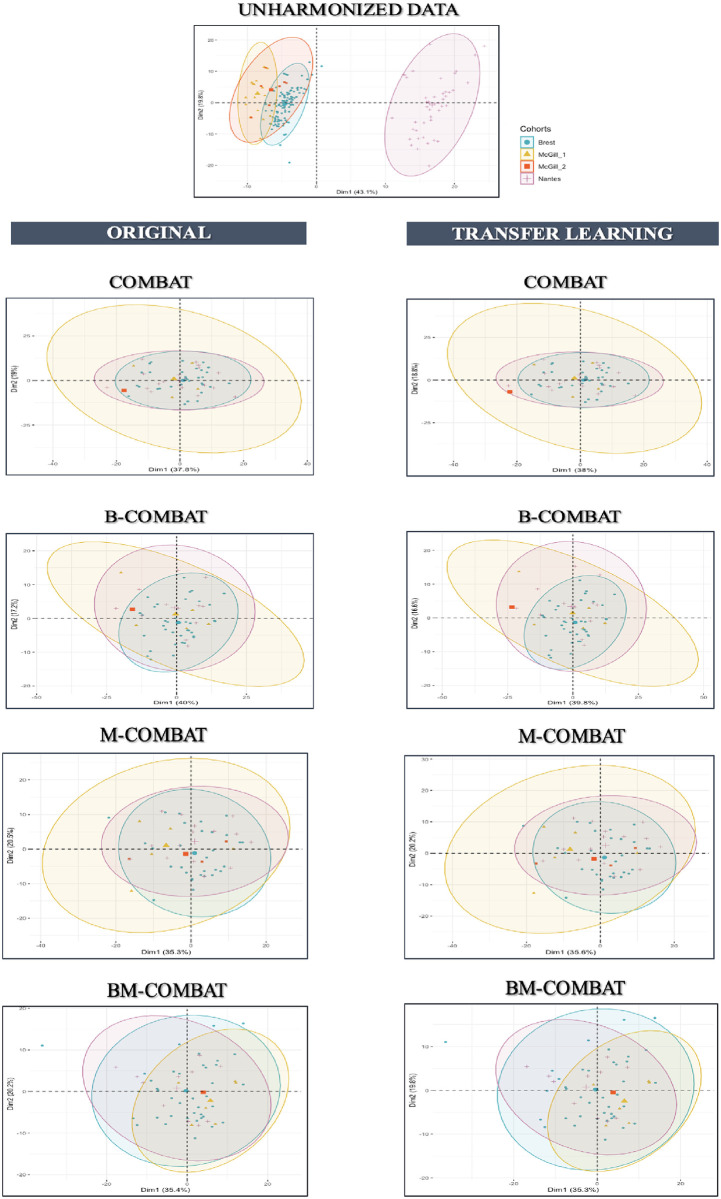
PCA and summary distribution in Experiment 1 in FDG PET. *Scatter plots of top 2 principal components of the radiomic features across the three labels (centers) using data transformed with the 4 versions of ComBat (using R (3*.*5*.*1) and R Studio (1*.*1*.*456*,*R Studios Inc*., *Boston*,*MA)* https://cran.r-project.org/).

Scatterplots of the top two principal components of PCA (Figs 3 and 4, representing ~54% and ~53% of the information in Orig and TL, respectively) confirm visually the ability of all four ComBat versions in removing the differences in radiomic features between labels while shifting the data to different locations. In the case of TL scenario, it shows that the method is rather effective in eliminating confounding effects brought by aforementioned variabilities.

### Predictive modelling using machine learning approaches

Table 3 provides results for the 3 performance evaluation metrics in the testing sets, for considering the use of the different ML algorithms in combination with the two different testing datasets, using the 4 versions of ComBat. The same results (including training sets and additional evaluation metrics) are provided in S1–S3 Figs.

**Table 3:** Performance metrics evaluation of predictive models in testing sets using the three ML pipelines.

ML	Data	AUC [0,1]		BAcc (%)		MCC [-1,+1]	
		Original	TL	Original	TL	Original	TL
**MR**	**Untransformed**	0.80	0.80	79	79	0.48	0.48
	**ComBat**	0.86	0.86	84	83	0.64	0.58
	**B-ComBat**	0.89	0.89	87	88	0.76	0.71
	**M-ComBat**	0.89	0.83	83	87	0.71	0.73
	**BM-ComBat**	0.90	0.91	85	88	0.73	0.78
**RF**	**Untransformed**	0.84	0.84	82	82	0.67	0.67
	**ComBat**	0.91	0.90	87	86	0.73	0.70
	**B-ComBat**	0.93	0.94	91	92	0.82	0.86
	**M-ComBat**	0.90	0.92	88	88	0.81	0.83
	**BM-ComBat**	0.93	0.95	93	94	0.86	0.90
**SVM**	**Untransformed**	0.79	0.79	77	77	0.61	0.61
	**ComBat**	0.85	0.83	81	80	0.66	0.65
	**B-ComBat**	0.88	0.88	83	84	0.73	0.75
	**M-ComBat**	0.90	0.89	83	81	0.70	0.76
	**BM-ComBat**	0.91	0.93	85	86	0.73	0.79

Original = original harmonized data (gold standard reference), TL = harmonized data by the proposed transfer learning method.

The experiment involves stratified random sampling used to split the data from the 3 centers into training and testing sets. In both sets, patients from the 3 centers are thus present. For the purpose of evaluating the TL ComBat harmonization, all patients from the testing set are not used in the initial harmonization. The results indicated in Table 3 show that all machine learning approaches led to models with good predictive performance (AUC 0.84–0.95, BAcc 81–93% and MCC 0.64–0.86), in the gold standard reference. All ComBat harmonized sets of features allowed for better models than using the untransformed data. In the TL scenario, the new patients (a set containing patients from all 3 centers) could be harmonized efficiently based on the learning of the harmonization transform, as the performance of the resulting model is very similar to the one of the models obtained using all the available data: between -0.06 to +0.02 in AUC, -2 to +4% in BAcc and -0.06 to +0.06 in MCC. Consistent with the gold reference, the absolute increase in performance between the use of the original, untransformed features and the harmonized ones utilizing the TL approach changed slightly depending on the ML algorithms utilized and between the patient populations considered. Table 4 confirms a statistically significant improvement for the three ML classification methods after harmonization compared to the use of untransformed features (in both *Original* and *TL*, respectively). Moreover, Table 4 also shows the lack of significant difference between the performance of ML models in original *vs*. TL.

**Table 4:** P-values of Welch’s t-test comparing ML algorithms performances on different 4 ComBat versions using original and TL data.

ML	Untransfomed vs. any ComBat	Original vs. Transfer learning			
		ComBat	B-ComBat	M-ComBat	BM-ComBat
MR	<0.0001	0.15	0.13	0.16	0.15
RF	<0.0001	0.14	0.12	0.15	0.13
SVM	<0.0001	0.13	0.12	0.15	0.13

## Discussion

Variations of scanner models, reconstruction algorithms and acquisition protocols are frequently unavoidable in multicenter studies, just as in retrospective studies with a long enlistment duration (*e*.*g*., when the scanner is replaced by one more model at some point). In such a context, there is a clear need for harmonization in order to allow for efficient models to be trained and validated. There are two main approaches to address this issue: (i). harmonizing images (i.e., before extracting features) and (ii). harmonizing features (i.e., *posteriori*, after their extraction). The first method tackles the issue in the image domain and early developed approaches considered standardization of acquisition protocols and reconstruction settings, relying on guidelines already available, e.g., for PET/CT imaging [43,44]. However, it has been shown recently that although such an approach can help towards reducing multicentre effects, it may still be insufficient to fully compensate them [43,45]. Techniques based on deep learning (convolutional neural networks, CNN or generative adversarial networks, GAN and their variants) have also been considered in order to standardize or harmonize medical images [44,46–48], including with an evaluation of the impact on resulting radiomic features, in the context of lung lesions in CT images [46]. Another paper [49] showed in a proposed workflow evaluating harmonization techniques using synthetic and real data comparing ComBat and cycleGaN that both methods perform well for removing various types of noises while preserving manually added synthesis lesions, but also for removing site effects on data coming from 2 different sites while preserving biological information. These techniques are promising but do not appear mature enough yet to enable a full comparison with the harmonization in the feature’s domain. Thus, as our team is currently developing such methods [50], we will definitively carry out such comparisons in future studies.

The other approach addresses the issue in the feature domain. This can be done either i) by selecting features before the statistical analysis based on their robustness, in order to eliminate features too sensitive to multicentre variability, or ii) by retaining all features together with their harmonizing their statistical properties so they can be grouped throughout the modeling step [20]. Numerous statistical methods exist to perform such normalization or batch-effect correction [20,26]. ComBat recently outperformed 6 other methods for batch effect removal using microarray datasets from brain RNA samples and two simulated datasets [25]. Although an extensive comparison of ComBat with other methods remains to be carried out explicitly in the context of radiomics, it has already been identified as a promising technique and is being increasingly and successfully used in recent radiomics studies [15,20,22,24,32,51–56]. It however has some limitations regarding its use in practice and we previously addressed two of these with the proposed BM-ComBat to allow for more flexibility in choosing a reference label and improving the estimation [44]. As expected and similarly to previous findings [44], in the experiment, all versions of ComBat were able to remove the differences amongst radiomic features and improve the predictive performance of the models, and the best results were consistently obtained with B(M)-ComBat over the standard ComBat, whether in the context of Original or TL scenarios.

The magnitude of differences between the performance of models trained and evaluated either in the original or the TL scenario is similar to the differences observed in a given scenario between different ML approaches. This absolute difference in performance amongst the ML algorithms can be attributed in part to i) the different feature selection techniques [41] and ii) the way the classifiers combine selected features. Previous studies have also highlighted the variability of resulting performance of radiomic models depending on either classifier or feature selection algorithms [20,57,58].

We proposed and evaluated a transfer learning modification to the well-known ComBat methodology for eliminating center-effects that allowed transferring the previously learned harmonization transform to the radiomic features based signatures values of new patients from a known center. Principal components analysis, analysis of variance, and statistical tests have shown the feasibility of this proposed harmonization approach, in the sense that the efficiency of the harmonization and the resulting performance of trained models in testing dataset is similar with the proposed TL approach, compared to the reference gold standard using all available data for the harmonization. These demonstrated that the proposed TL technique leads to efficient estimation with similar resulting predictive ability of models. This important point was demonstrated across 3 different ML algorithms, all performance metrics and for both experiments. More specifically, the experiment showed that the TL approach was effective in applying the previously determined harmonized transform to the radiomic features values of new patients from a known center resulting in a consistent improvement in the predictive performance of the developed models. Although the proposed TL technique provided a consistent comparable predictive performance of the developed models in different ML algorithms, we acknowledge the limitations associated with relatively small improvements in combination with a single dataset with limited heterogeneity in the imaging factors. We also performed a single split of the data and did not investigate different combinations of training/testing with the 3 available centers, due to the time-consuming building of numerous models for evaluation. Future work could consider different splits and combination of centers. The proposed method will thus require validation in larger and more diverse cohorts (more centers, more scanners and sources of variability). Our future work will thus consider the use of a small set of patients from the entirely new center as examples to learn from, in order to improve the performance of the proposed TL approach in this context. In addition, our proposed approach does not alleviate one of its inherent limitations: (i) ComBat only works properly when available and labelled data is available in order to perform the estimate and batch correction, (ii) in order to apply the developed/validated model (*i*.*e*., a combination of harmonized radiomic features with an associated threshold value) to a new patient from another center not previously included, there is currently no direct method to apply the previously determined harmonization transform to the radiomic features values of this new patient in order to determine his/her prediction.

Finally, as in our previous work, we have considered working with the entire set of radiomic features irrespectively of their robustness (i.e., without first selecting features based on their resilience to changes in reconstruction or acquisition settings). Identifying radiomic features robust to changes in acquisition and reconstruction settings prior to feeding them to the machine learning pipeline is also a different approach. Such a feature selection procedure can help building more robust models, potentially without the need for harmonization, since only features insensitive (or at least, less sensitive) to multicenter variability are therefore exploited. This approach however may suffer from a potential loss of information, as features identified as unreliable are usually discarded before being evaluated and the most robust/reproducible features might not necessarily be the most clinically-relevant to the task at hand. Furthermore, the size of the radiomic features set would depend on the chosen threshold of what is considered robust enough. We will compare such an approach with ComBat harmonization in our future works.

None of the models predicting local failure selected shape features and they relied only on intensity and textural ones (whether considering the untransformed or the harmonized features), which indicates that at least in that application, the shape and size of the tumor in PET and MRI is not informative, as already observed in our initial studies in that cohort [32,59]. Shape features can be expected to be less impacted by the center effect, compared to intensity and textural features, especially since the delineation was the same for all images. However, they might still be sensitive to factors such as spatial resolution (more or less blur at the edges of the tumors will drive more or less complex shapes and surfaces) and voxel sampling (larger voxels will lead to less detailed delineations and “simpler” shapes and surfaces). Indeed, distributions of most shape features were found to be statistically different between the 3 MRI or the 4 PET batches, although the statistics was lower than for intensity and textural features.

Finally, it could also be interesting to investigate the feasibility to apply this transfer learning approach in a different implementation framework such as distributed learning [60,61].

## Conclusion

The transfer learning technique implemented within ComBat allowed applying the previously determined harmonization transform to the radiomic features values of new patients from a known center with a slightly stronger decrease in performance. Our approach alleviates one of the most important limitations of ComBat for harmonization of radiomic features in a multicentre context when new, previously unseen data are to be analyzed.

## Supporting information

S1 Fig Performance metrics evaluation of predictive models using MR with LASSO. (TIFF)

S2 Fig Performance metrics evaluation of predictive models using RF. (TIFF)

S3 Fig Performance metrics evaluation of predictive models using SVM. (TIFF)

S1 Table Patient’s characteristics. (PDF)

S2 Table PET/CT and MRI protocols in Brest (A), Nantes (B), and McGill (C). (PDF)

S1 Data (CSV)
