# Supplementary figures and images for: A transfer learning approach to facilitate ComBat-based harmonization of multicentre radiomic features in new datasets
Source: PLoS One. 2021 Jul 1;16(7):e0253653. doi: 10.1371/journal.pone.0253653 (PMC8248970; doi:10.1371/journal.pone.0253653)

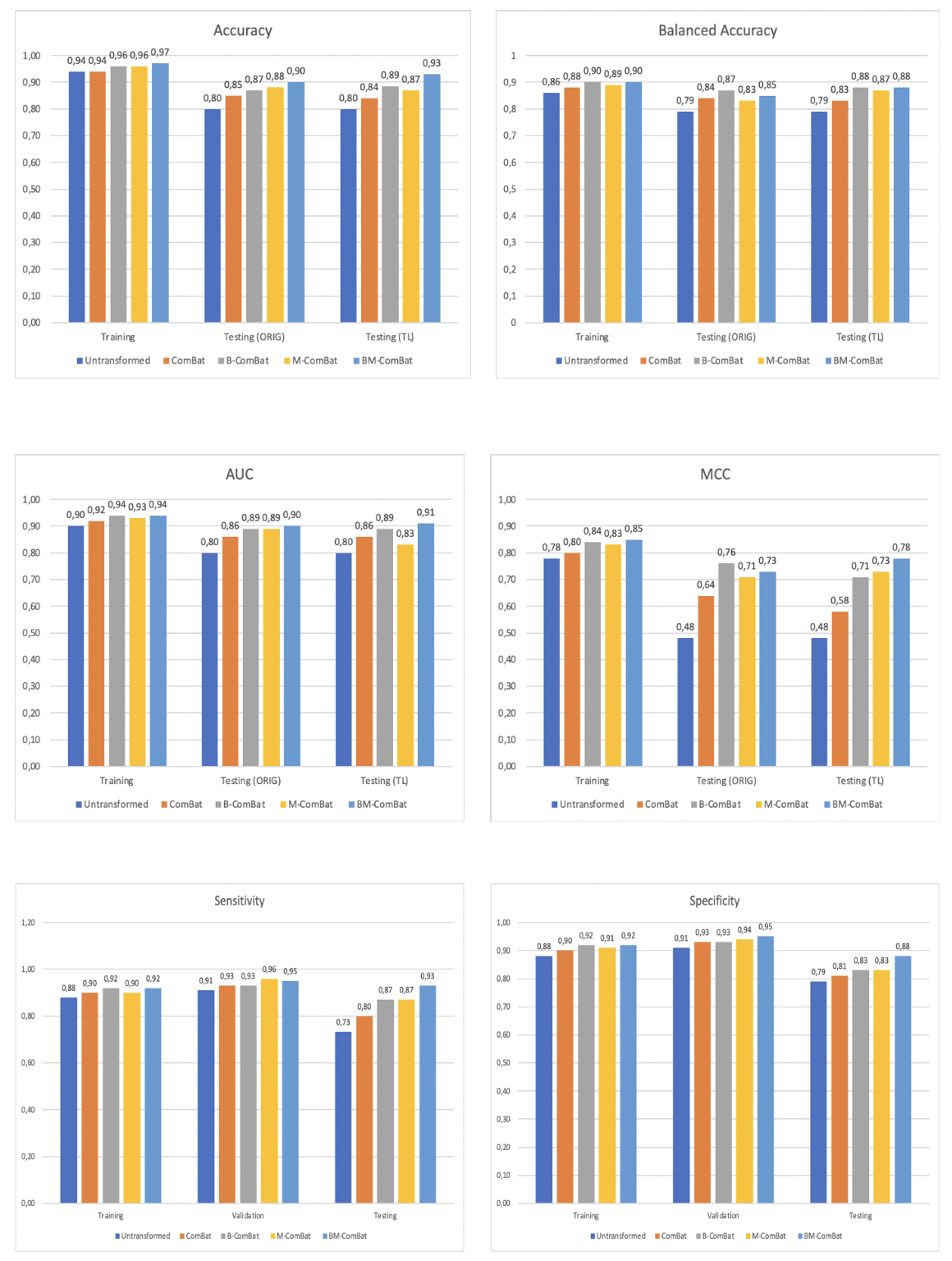

Supplement: S1 Fig — (TIFF) [file pone.0253653.s001.tiff]

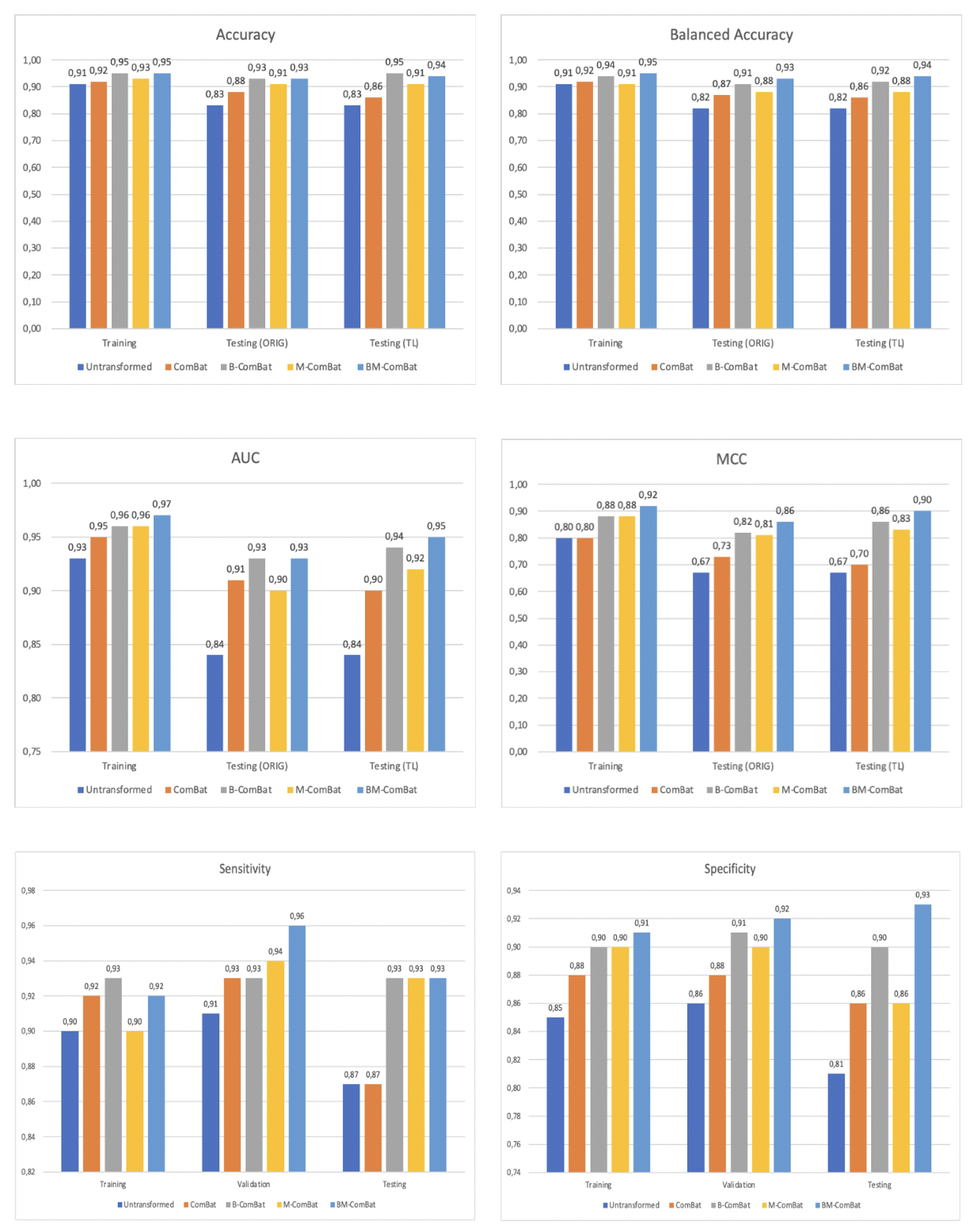

Supplement: S2 Fig — (TIFF) [file pone.0253653.s002.tiff]

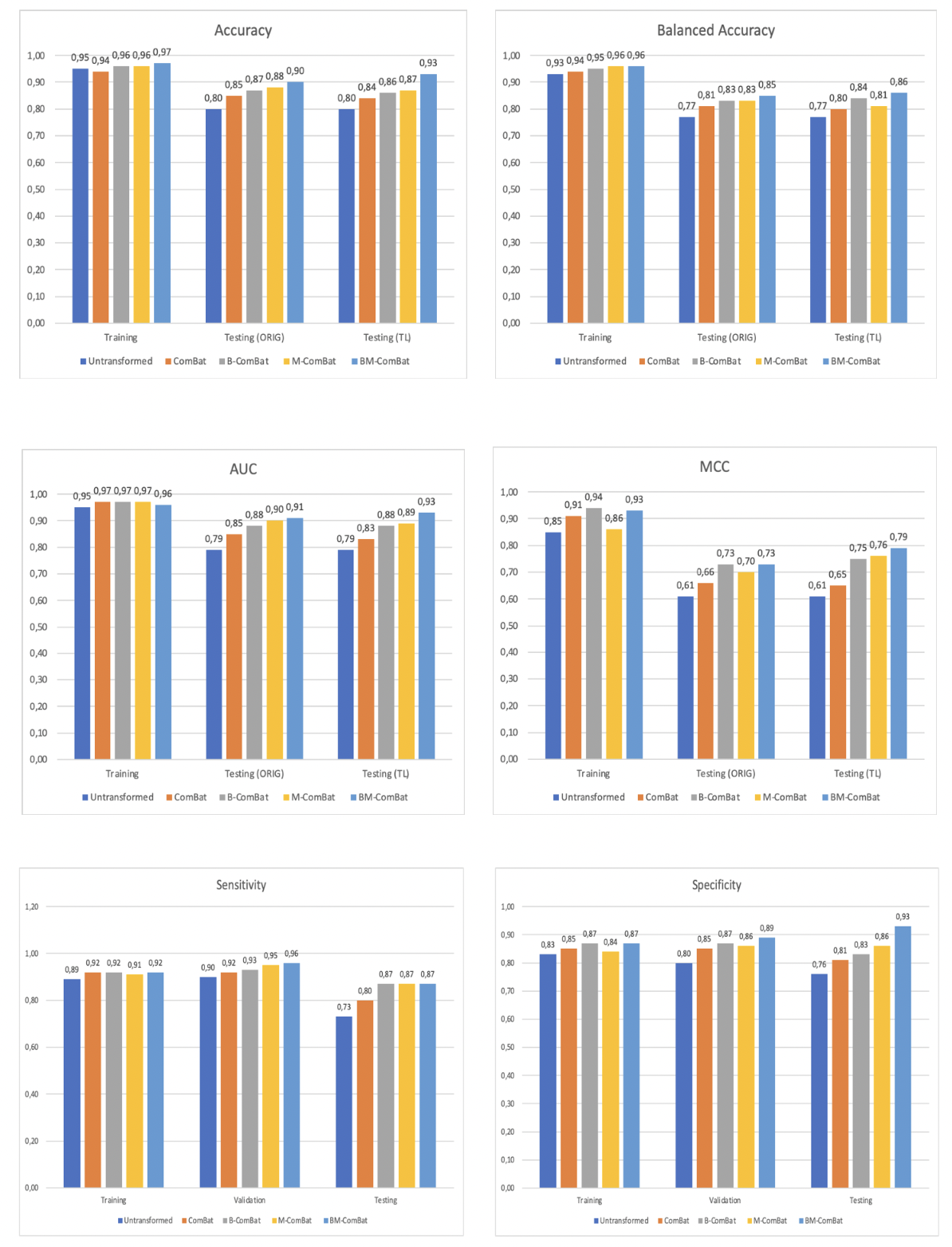

Supplement: S3 Fig — (TIFF) [file pone.0253653.s003.tiff]
