# Supplementary material for: A transfer learning approach to facilitate ComBat-based harmonization of multicentre radiomic features in new datasets
Source: PLoS One. 2021 Jul 1;16(7):e0253653. doi: 10.1371/journal.pone.0253653 (PMC8248970; doi:10.1371/journal.pone.0253653)
Supplement: S1 Table — (PDF) [file pone.0253653.s004.pdf]

Characteristics	Patients		
	**Brest**	**Nantes**	**McGill**
	n=117	*n=44*	*n=28*
Age median (range)	58 (29-90)	51 (23-73)	52 (26-86)
**FIGO Stage**			
IB1	3	1	0
IB2	11	4	4
IIA1	7	3	0
IIB	61	27	14
IIIA	2	1	1
IIIB	18	3	7
IVA	15	3	2
IVB			
**Histology**			
Squamous	94	40	24
Adenocarcinoma	17	1	1
Adenosquamous carcinoma	1	0	0
Clear cell carcinoma	5	3	3
**Lymph node involvement**			
Uninvolved	55	23	7
Involved	57	27	21
Pelvic	40	19	16
Pelvic and para-aortic	17	8	5
**CBC median (range)**			
White blood cells	8.1 10^3/ml (4.6 – 25.6)	8.1 10^3/ml (4.6 – 25.6)	X
Hemoglobin	127 g/dl (71 – 151)	123 g/dl (67 – 147)	X
Platelets	257.3 10^3/ml (14 – 42)	273.9 10^3/ml (152 – 767)	X
Body mass index median (range)	23.1 (14 – 42)	22.7 (13 – 39)	X
**Treatment**			
3D-CRT	62	2	4
IMRT	55	42	24
EBRT dose median (range)	45 (45 – 54)	45 (45 – 54)	45 (45 – 54)
BT dose median (range)	29 (21 – 28)	29 (21 – 28)	29 (21 – 28)
Overall treatment time (range)	49 (48 – 53)	49 (48 – 53)	49 (48 – 53)

FIGO International Federation of Gynecology and Obstetrics, CBC complete blood counts, 3D-CRT three-dimensional conformal radiotherapy, IMRT intensity-modulated photon radiotherapy, EBRT external beam radiotherapy, BT brachytherapy

S1 Table. Patient's characteristics.
