# Supplementary material for: A transfer learning approach to facilitate ComBat-based harmonization of multicentre radiomic features in new datasets
Source: PLoS One. 2021 Jul 1;16(7):e0253653. doi: 10.1371/journal.pone.0253653 (PMC8248970; doi:10.1371/journal.pone.0253653)
Supplement: S2 Table — (PDF) [file pone.0253653.s005.pdf]

S2 Table. PET/CT and MRI protocols in Brest (A), Nantes (B), and McGill (C)

(A)

	Preparation	Technical characteristics
PET	Patients fasted for 4h before acquisition<br>The blood glucose level had to be less than 7 mmol/L<br>Injection of 5 MBq/kg of ^{18}F-FDG<br>PET acquisitions were carried out approximately 60min after injection	Routine clinical image reconstruction protocols were used: for the Philips GEMINI, data were reconstructed using the RAMLA 3D (2 iterations, relaxation parameter 0.05) whereas for the Siemens Biograph, images were reconstructed with Fourier rebinning (FORE) followed by OSEM (2 iterations, 8 subsets). In both cases images were corrected for attenuation using the corresponding CT, reconstructed with a 2×2×2 mm^3 voxels grid and post-filtered with a 5-mm FWHM 3D Gaussian.
CT	N/A (acquired with the PET/CT acquisition)	The CT consisted of a 64-slice multidetector-row spiral scanner with a transverse field of view of 700 mm. Standard CT parameters were used: a collimation of 16×1.2 mm^2, pitch 1, tube voltage of 120 kV, and effective tube current of 80 mA.

Sequences	Plane	Technical characteristics
T2-w	Axial (renal hilum-pubis)	Axial: TR=3425ms, TE=110ms, NSA: 2, ST/G: 4.5/1, matrix: 340×350, FOV: 38, AT=3.30 min
	Sagittal	Sagittal: TR=3425ms, TE=110ms NSA: 3, ST/G: 3.5/1.2, matrix: 348×276, FOV: 25, AT=3.36 min
	Axial oblique (perpendicular to cervical axis or/and along with endometrial cavity axis)	Axial oblique: TR=3425ms, TE=110ms, NSA: 6, ST/G: 4/0.4, matrix: 256/176, FOV: 18, AT=3.30 min
T1-w	Axial (renal hilum-pubis)	TR=575ms, TE=7.7 to 17ms, NSA: 1, ST/G: 6/2, matrix: 300×205, FOV: 36, AT=2.16 min

	Axial and sagittal	
T1-FS+CE	All except two allergic patients (training set) received a 0.1mmol/kg injection of gadobenate dimeglumine (Multihance; Bracco Diagnostics, Milan, Italy).	TR=540ms, TE=10 to 12ms, NSA: 2, ST/G: 4.5/1, matrix: 360×252, FOV: 38, AT=3.28 min
DWI	Axial oblique and sagittal, b value=(0, 400, 1000) s/mm² ADC maps creation: For each acquisition, the ADC was computed voxel by voxel as the slope of the linear regression of the logarithm of the DWI exponential signal decay on the three b-values.	TR=3900ms, TE=80ms NSA: 12, ST/G: 6/0 matrix: 124×174, FOV: 35, AT=3.40 min

Abbreviations: T2-W: T2-weighted, T1-W: T1-weighted, T1-FS+CE: T1 fat-suppressed with contrast enhancement, DWI: diffusion-weighted imaging,
AT: acquisition time, TR: repetition time, TE: echo time, NSA: number of signal acquisition, ST (mm): slice thickness, G (mm): gap, FOV (cm): field of view (right to left).

(B)

	Preparation	Technical characteristics
PET	Patients fasted for 4h before acquisition The blood glucose level had to be less than 7 mmol/L Injection of 5 MBq/kg of ^{18}F-FDG PET acquisitions were carried out approximately 60min after injection	Routine clinical image reconstruction protocols were used: for the Siemens Biograph, images were reconstructed with Fourier rebinning (FORE) followed by OSEM (2 iterations, 8 subsets). Images were corrected for attenuation using the corresponding CT, reconstructed with a 2×2×2 mm^3 voxels grid and post-filtered with a 5-mm FWHM 3D Gaussian.
CT	N/A (acquired with the PET/CT acquisition)	The CT consisted of a 64-slice multidetector-row spiral scanner with a transverse field of view of 700 mm. Standard CT parameters were used: a collimation of 16×1.2 mm^2, pitch 1, tube voltage of 120 kV, and effective tube current of 80 mA.

Sequences	Plane	Technical characteristics
T2-w	Axial (renal hilum-pubis)	Axial: TR=3445ms, TE=105ms, NSA: 2, ST/G: 4.5/1, matrix: 384×384, FOV: 38, AT=3.25 min

	Sagittal	Sagittal: TR=3425ms, TE=110ms NSA: 3, ST/G: 3.5/1.2, matrix: 348×276, FOV: 25, AT=3.25 min
	Axial oblique (perpendicular to cervical axis or/and along with endometrial cavity axis)	Axial oblique: TR=3445ms, TE=105ms, NSA: 6, ST/G: 4/0.4, matrix: 276x194, FOV: 18, AT=3.25 min
T1-w	Axial (renal hilum-pubis)	TR=575ms, TE=8.5 to 16ms, NSA: 1, ST/G: 6/2, matrix: 320×240, FOV: 36, AT=2.16 min
T1-FS+CE	Axial and sagittal<br>All except two allergic patients (training set) received a 0.1mmol/kg injection of gadolinium chelate (gadoteric acid; Dotarem Guerbet, Aulnay-sous-Bois, France).	TR=560ms, TE=10 to 12ms, NSA: 2, ST/G: 4.5/1, matrix: 320×240, FOV: 38, AT=3.22 min
DWI	Axial oblique and sagittal, b value=(0, 400, 1000) s/mm²<br>ADC maps creation: For each acquisition, the ADC was computed voxel by voxel as the slope of the linear regression of the logarithm of the DWI exponential signal decay on the three b-values.	TR=3800ms, TE=80ms NSA: 12, ST/G: 6/0 matrix: 148×192, FOV: 35, AT=3.30 min

Protocole 1
Abbreviations: T2-W: T2-weighted, T1-W: T1-weighted, T1-FS+CE: T1 fat-suppressed with contrast enhancement, DWI: diffusion-weighted imaging,
AT: acquisition time, TR: repetition time, TE: echo time, NSA: number of signal acquisition, ST (mm): slice thickness, G (mm): gap, FOV (cm): field of view (right to left).

Sequences	Plane	Technical characteristics
T2-w	Axial (renal hilum-pubis)	Axial: TR=3435ms, TE=105ms, NSA: 2, ST/G: 4.5/1, matrix: 384×384, FOV: 38, AT=3.30 min
	Sagittal	Sagittal: TR=3435ms, TE=110ms NSA: 3, ST/G: 3.5/1.2, matrix: 348×276, FOV: 25, AT=3.35 min
	Axial oblique (perpendicular to cervical axis or/and along with endometrial cavity axis)	Axial oblique: TR=3435ms, TE=105ms, NSA: 6, ST/G: 4/0.4, matrix: 256x194, FOV: 18, AT=3.25 min

T1-w	Axial (renal hilum-pubis)	TR=575ms, TE=9 to 15ms, NSA: 1, ST/G: 6/2, matrix: 320×240, FOV: 36, AT=2.16 min
T1-FS+CE	Axial and sagittal<br><br>All except two allergic patients (training set) received a 0.1mmol/kg injection of gadolinium chelate (gadoteric acid; Dotarem Guerbet, Aulnay-sous-Bois, France).	TR=550ms, TE=10 to 12ms, NSA: 2, ST/G: 4.5/1, matrix: 320×256, FOV: 38, AT=3.22 min
DWI	Axial oblique and sagittal, b value=(0, 400, 1000) s/mm²<br><br>ADC maps creation: For each acquisition, the ADC was computed voxel by voxel as the slope of the linear regression of the logarithm of the DWI exponential signal decay on the three b-values.	TR=3750ms, TE=80ms NSA: 12, ST/G: 6/0 matrix: 156×196, FOV: 35, AT=3.30 min

Protocole 2
Abbreviations: T2-W: T2-weighted, T1-W: T1-weighted, T1-FS+CE: T1 fat-suppressed with contrast enhancement, DWI: diffusion-weighted imaging,
AT: acquisition time, TR: repetition time, TE: echo time, NSA: number of signal acquisition, ST (mm): slice thickness, G (mm): gap, FOV (cm): field of view (right to left).

(C)

	Preparation	Technical characteristics
PET	Patients fasted for 4h before acquisition<br>The blood glucose level had to be less than 7 mmol/L<br>Injection of 8.14 MBq/kg of ^{18}F-FDG<br>PET acquisitions were carried out approximately 60min after injection	Routine clinical image reconstruction protocols were used: for the Discovery ST, images were reconstructed with OSEM (2 iterations, 8 subsets). Images were corrected for attenuation using the corresponding CT, reconstructed with a 3.65×3.65×3.27 mm^3 voxels grid and post-filtered with a 5-mm FWHM 3D Gaussian.
CT	N/A (acquired with the PET/CT acquisition)	The CT consisted of a 16-slice multidetector-row spiral scanner with a transverse field of view of 700 mm. Standard CT parameters were used: a collimation of 16×1.2 mm^2, pitch 1, tube voltage of 140 kV, and effective tube current of 90 mA.

Sequences	Plane	Technical characteristics
T2-w	Axial (renal hilum-pubis)	Axial: TR=4575ms, TE=100ms, NSA: 4, ST/G: 4/0, matrix: 512×256, FOV: 24, AT=3.25 min
	Sagittal	Sagittal: TR=4575ms, TE=100ms, NSA: 4, ST/G: 4/0, matrix: 512×256, FOV: 24, AT=3.25 min
	Axial oblique (perpendicular to cervical axis or/and along with endometrial cavity axis)	Axial oblique: TR=4000ms, TE=100ms, NSA: 4, ST/G: 4/0, matrix: 512×256, FOV: 24, AT=3.25 min
T1-w	Axial (renal hilum-pubis)	TR=565ms, TE=9 to 11ms, NSA: 1, ST/G: 4/0, matrix: 320×192, FOV: 26, AT=3.26 min
T1-FS+CE	Axial and sagittal<br>All received a 0.1mmol/kg injection of gadolinium chelate (Gadovist; Bayer, Canada).	TR=3.6ms, TE=1.75ms, NSA: 1, ST/G: 4/0, matrix: 320×192, FOV: 26, AT=3.26 min
DWI	Axial oblique and sagittal, b value=(0, 500, 1000) s/mm²<br>ADC maps creation: For each acquisition, the ADC was computed voxel by voxel as the slope of the linear regression of the logarithm of the DWI exponential signal decay on the three b-values.	TR=5000ms, TE=69ms NSA: 8, ST/G: 6/0 matrix: 128×256, FOV: 32, AT=3.35 min

Abbreviations: T2-W: T2-weighted, T1-W: T1-weighted, T1-FS+CE: T1 fat-suppressed with contrast enhancement, DWI: diffusion-weighted imaging,
AT: acquisition time, TR: repetition time, TE: echo time, NSA: number of signal acquisition, ST (mm): slice thickness, G (mm): gap, FOV (cm): field of view (right to left).
